# Dissociation kinetics of small-molecule inhibitors in Escherichia coli is coupled to physiological state of cells

**DOI:** 10.1038/s42003-023-04604-9

**Published:** 2023-02-25

**Authors:** Dai Le, Tatsuya Akiyama, David Weiss, Minsu Kim

**Affiliations:** 1 Department of Physics, Emory University, Atlanta, GA 30322 USA; 2 Graduate Division of Biological and Biomedical Sciences, Emory University, Atlanta, GA 30322 USA; 3 Division of Infectious Diseases, Department of Medicine, Emory University, Atlanta, GA 30322 USA; 4 Antibiotic Research Center, Emory University, Atlanta, GA 30322 USA

**Keywords:** Antimicrobials, Drug discovery, Biophysics

## Abstract

Bioactive small-molecule inhibitors represent a treasure chest for future drugs. In vitro high-throughput screening is a common approach to identify the small-molecule inhibitors that bind tightly to purified targets. Here, we investigate the inhibitor-target binding/unbinding kinetics in E. coli cells using a benzimidazole-derivative DNA inhibitor as a model system. We find that its unbinding rate is not constant but depends on cell growth rate. This dependence is mediated by the cellular activity, forming a feedback loop with the inhibitor’s activity. In accordance with this feedback, we find cell-to-cell heterogeneity in inhibitor-target interaction, leading to co-existence of two distinct subpopulations: actively growing cells that dissociate the inhibitors from the targets and non-growing cells that do not. We find similar heterogeneity for other clinical DNA inhibitors. Our studies reveal a mechanism that couples inhibitor-target kinetics to cell physiology and demonstrate the significant effect of this coupling on drug efficacy.

## Abstract (web summary)

This study investigates how binding/unbinding kinetics of a benzimidazole-derivative DNA inhibitor in E. coli cells depends on cell growth and physiology and demonstrates the effect of this coupling on drug efficacy.

## Introduction

Bioactive small-molecule inhibitors constitute the majority of drugs on the market. However, identifying efficacious lead compounds and developing them as new drugs is a time-consuming and expensive process, which typically requires more than a decade and billions of dollars^1^. Even these staggering numbers are likely an underestimate, because they do not fully account for numerous failures at early stages^2^. A major consequence of these inefficiencies is the shortage of new drugs, particularly in the anti-bacterial arsenal. This shortage in light of rising bacterial resistance is a serious medical problem.

In recent years, target-based drug screening has become a central paradigm to streamline the drug discovery process^3,4^. By screening vast compound libraries against macromolecules essential for cellular function in vitro, target-based screening rapidly identifies a wealth of small molecule inhibitors. Unfortunately, many of these candidates turn out to be therapeutically ineffective and are abandoned after substantial investment^5,6^. One major issue of this screening is that it is performed with solutions reconstituted with purified targets^7–11^. In reality, however, the targets reside in/on cells and interact with the inhibitors in highly complex cellular environments. Currently, little is known about how this natural cellular environment affects the inhibitor-target binding/unbinding kinetics. The present study aims to reduce this gap by characterizing the inhibitor-target kinetics in live bacterial cells.

Benzimidazole is one of the most frequent moieties in therapeutic drugs listed by the US Food and Drug Administration^12,13^. It is being extensively utilized in medicinal chemistry as a promising scaffold for new drug design^14–19^, because it exhibits high affinity to nucleotides. DNA is a particularly important drug target. DNA-binding inhibitors have been successfully adopted as anticancer drugs^20–22^ and are being tested to treat other human diseases, including malaria and HIV^23–27^. Recently, DNA inhibitors have received increasing attention as anti-bacterial drugs due to the rising bacterial resistance^28–32^.

A benzimidazole derivative known as Hoechst 33342 (HCT)^33^ binds to DNA to inhibit gene expression^34^. In this study, HCT was used as a model system because HCT fluoresces only when bound to DNA and loses fluorescence upon unbinding^35–38^, which allows us to directly monitor the binding and unbinding kinetics. Our data indicate that the rate of unbinding is not constant but is strongly correlated with the rate of cell growth, which was mediated by transcription. This correlation, together with the known mode of action of HCT, suggests a double negative feedback loop between HCT-DNA complex formation and gene expression. In accordance with this loop, we observed a great deal of heterogeneity in the accumulation of HCT on its target, which leads to heterogeneous growth inhibition. These heterogeneous effects were observed for other DNA inhibitors as well.

## Results

### The rate of HCT unbinding from DNA varies with the HCT concentration

It was previously shown that HCT could fluoresce when embedded in the cell membrane^39^. However, HCT fluorescence signal arising from the interaction with cell membrane is expected to be weak^40^. To test this, we co-labeled DNA in live E. coli cells with HCT and mCherry-tagged DNA-associated protein HupA (HupA-mCherry)^41^. The spatial profiles of their intensities were identical, which demonstrates that the majority of the HCT signal in cells comes from its binding to DNA (Supplementary Fig. 1).

**Fig. 1:**
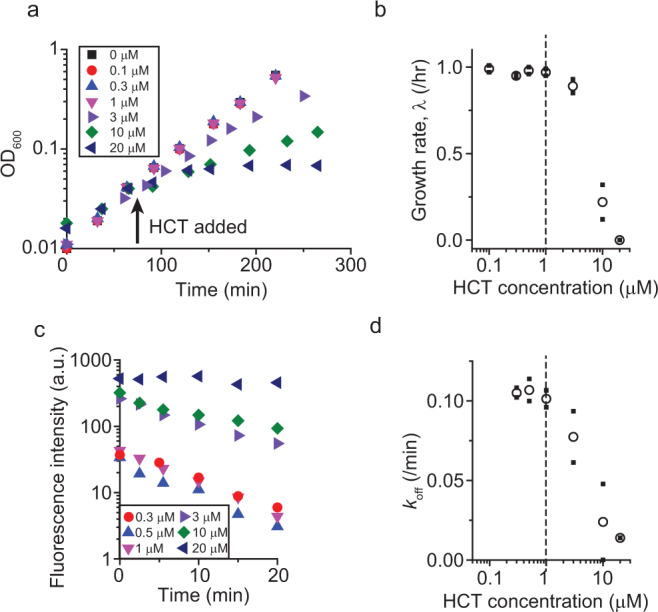
*λ* and *k*_off_ at various HCT concentrations. **a**, **b** HCT slows down the growth of E. coli cells in bulk culture at concentrations above 1 μM. A *t*-test (one-tailed) between the growth rates for 1 and 3 μM shows a *P*-value equal to 0.0325. Therefore, their difference is considered to be statistically significant. This agrees with a noticeable and persistent difference of OD_600_ between 1 and 3 μM treatment cultures (**a**). **c**, **d** Cells were pre-incubated with HCT and suspended in HCT-free media (which defines time zero). For each data point, the intracellular HCT intensities of ~150 cells were measured and averaged. The slope of the intensity change reflects the *k*_off_. The *k*_off_ decreased at HCT concentrations above 1 μM. **a** and **c** show data from a single experiment. A biological replicate was conducted to confirm that the pattern was reproducible. Small solid symbols in **b** and **d** indicate the data from two biological repeats, and the open symbols indicate their mean.

We then characterized the inhibitory effects of HCT on E. coli cells by measuring the growth rate, *λ* (Fig. 1a, b). *λ* was unchanged up to 1 μM but decreased sharply at higher HCT concentrations (Fig. 1a, b). The repressive effect of HCT on cell growth is expected based on its ability to bind to DNA, which results in the inhibition of gene expression^34^. Indeed, when we measured the expression of a reporter gene (*lacZ*), we found that its expression was inhibited by HCT (Supplementary Fig. 2).

We then measured the binding and unbinding kinetic rates *k*_on_ and *k*_off_. To measure the *k*_on_, we exposed cells to HCT and monitored an increase in the intracellular HCT fluorescence intensity (Supplementary Fig. 3), as similarly done in previous in vitro studies^42,43^. The *k*_on_ was constant over a wide range of HCT concentrations tested (Supplementary Fig. 3). To measure the *k*_off_, we pre-incubated cells with HCT, resuspended them in HCT-free media, and measured a decrease in the intercellular HCT intensity (Fig. 1c), the slope of which is equal to the *k*_off_. We found that the *k*_off_ remained constant up to 1 μM HCT at ~0.1/min (Fig. 1c, d). This value is at least one order of magnitude lower than the *k*_off_ measured in vitro, which is in the range of 1~10/min^42,43^. The *k*_off_ in cells was even lower at higher HCT concentrations (Fig. 1c, d).

### Correlation between unbinding rate and growth rate

**Fig. 2:**
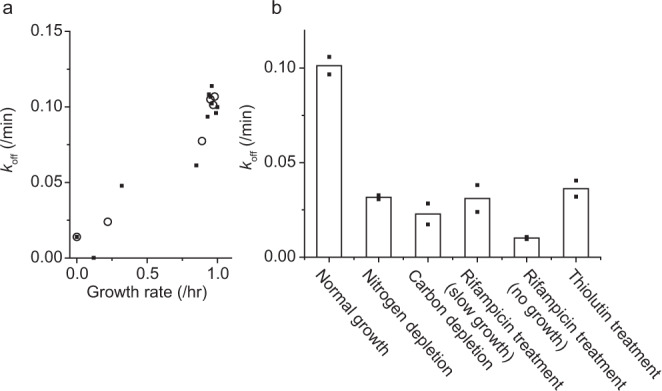
Cell growth affects HCT unbinding. **a** Data from Fig. 1b and d were put together to find the relationship between *k*_off_ and *λ*. **b** *k*_off_ under various conditions. Rifampicin and thiolutin were used to repress transcription. The results show an important effect of transcription on *k*_off_. See Supplementary Fig. 5 for *k*_off_ under translation repression by chloramphenicol. See Supplementary Videos 4 and 5 for time-lapse images of HCT intensity under rifampicin treatment and chloramphenicol treatments, respectively. Small solid symbols indicate the data from two biological repeats, and the open symbols and columns indicate their mean.

Interestingly, the threshold concentrations above which the *k*_off_ and *λ* decrease coincided (1 μM in Fig. 1b and d, dashed line), which suggests that the *k*_off_ and *λ* be coupled. This coupling can be more directly visualized by plotting *k*_off_ as a function of *λ*, which showed a strong correlation (Fig. 2a). While the HCT dilution due to cell growth, which includes the cell volume expansion and DNA synthesis, could alter *k*_off_, its effect (*λ* ≈ 1/hr = 0.017/min) is an order of magnitude less than a change we observed (Fig. 2a) and thus negligible.

A recent survey examined the common properties in approved drug molecules and showed that the drug-target unbinding kinetics are a major determinant of drug efficacy^44–46^. The correlation we observed agrees with this survey result. However, one important distinction should be made. Previously, the *k*_off_ of inhibitors was measured in test tubes against purified targets and considered as an intrinsic constant value. However, our data show that the *k*_off_ in cells is not constant but variable.

Motivated by the correlation between *λ* and *k*_off_, we next wondered how repressing cell growth with other means would affect unbinding. We thus quantified the *k*_off_ in cells whose growth was repressed by nutrient depletion, either nitrogen or carbon. For this experiment, 1 μM HCT was used; note this HCT concentration had little effects on cell growth (Fig. 1b). We found that the growth repression by nutrient depletion led to a decrease in the *k*_off_ (Fig. 2b).

Next, we repressed cell growth by inhibiting transcription. DNA stores genetic information, which is transcribed by RNA polymerases (RNAPs). RNAPs have high affinities for DNA and are processive^47–49^. Thus, when they encounter protein roadblocks bound to DNA, they dislodge these proteins and continue their functions. We wondered whether this processivity facilitated unbinding of DNA inhibitors. To test this, we measured the *k*_off_ in transcription-repressed cells. Rifampicin is known to repress transcription in cells. When cells were treated with rifampicin, it took some time for cell growth to slow down and completely stop (Supplementary Fig. 4). We found that the *k*_off_ was very low during this slow-down period and even lower when cell growth stopped (Fig. 2b). We repeated the experiment with thiolutin, another transcription repressor. Treatment of thiolutin led to immediate slow-down of cell growth (Supplementary Fig. 4). We again observed that the *k*_off_ decreased after thiolutin treatment.

This finding with transcription inhibition is consistent with the above measurement with nutrient-deprived cells. As cells are depleted of nutrients, the rate of transcription drops dramatically^50–52^, which can explain a low *k*_off_ in nutrient-depleted conditions (Fig. 2b). To further demonstrate the importance of transcription, we measured the *k*_off_ in cells treated with a translation repressor, chloramphenicol. We found that chloramphenicol treatment reduces the *k*_off_ only marginally (Supplementary Fig. 5), which further underscores the importance of transcription (but not translation) to the unbinding kinetics.

### Heterogeneous effects of DNA inhibitor on cell growth

**Fig. 3:**
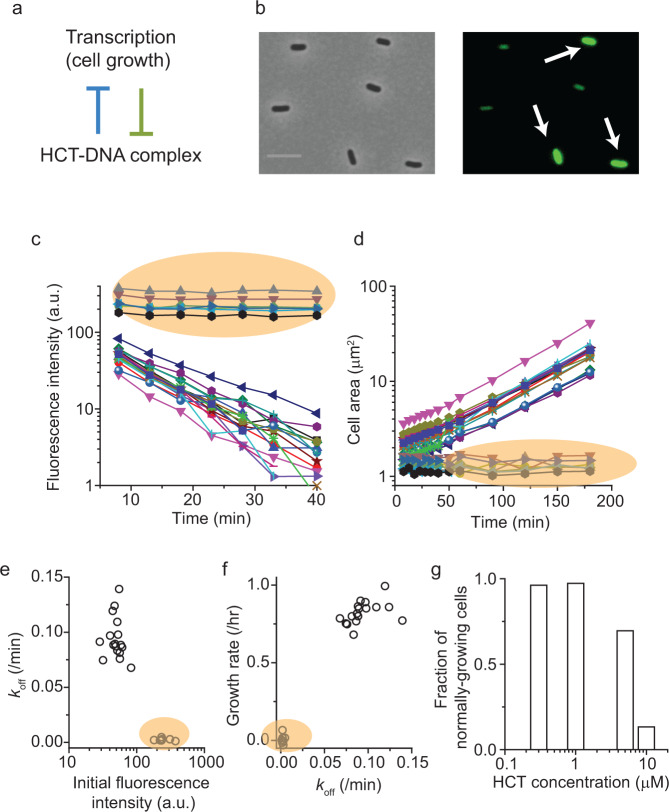
Heterogeneous inhibitory activities of HCT at single-cell resolution. **a** Formation of HCT-DNA complex represses transcription, while transcription represses the complex formation by stimulating unbinding. **b** Cells were pre-incubated with 5 μM HCT and imaged with a microscope at single-cell resolution. We found two distinct levels of intracellular HCT intensity, indicating that its accumulation on the target (DNA) is heterogeneous across a cell population. Note that HCT emits blue fluorescence but is depicted as green here for the clarity of the images. The scale bar represents 5 μm. **c**, **d** Cells pre-incubated with 5 μM HCT were transferred to HCT-free media (which defines time zero), and intracellular HCT intensity and cell area were observed over time using a microscope. Cells exhibiting high HCT intensity (orange shades) exhibited no HCT unbinding and no growth. On the other hand, cells with initially weak HCT intensity exhibited rapid HCT unbinding and active cell growth. **e**, **f** Data plotted in **c**, **d** were quantitatively analyzed. **g** The fraction of the subpopulation with rapid HCT unbinding and active cell growth decreases at higher external HCT concentrations. The plotted data were from a single experiment. A biological replicate was conducted to confirm the pattern.

When HCT (or other drug molecules) binds to DNA, the formation of HCT-DNA complex negatively affects the transcription (Supplementary Fig. 2 and ref. ^34^). This negative effect is depicted as a blue line in Fig. 3a. Our finding above shows that transcription negatively affects the formation of HCT-DNA by facilitating the unbinding (Fig. 2b); this effect is depicted as a green line in Fig. 3a. As a result, they constitute a double negative feedback loop, which is known to trigger bi-stability in a system^53^. In the present context, this bi-stability is represented as two subpopulations. In one subpopulation, the inhibitors would unbind from their targets rapidly, leading to low HCT accumulation on the targets. As a result, this subpopulation will exhibit active transcription and fast cell growth. In the other subpopulation, the slow unbinding of inhibitors would lead to higher HCT accumulation on the targets, leading to strong inhibition of transcription and slow (or no) cell growth.

We tested this hypothesis by measuring HCT levels and cell growth with time-lapse single-cell microscopy. In our population study above, we observed a moderate decrease in the *k*_off_ and *λ* at 5 μM HCT. Cells were pre-incubated with this concentration and imaged with a microscope. We observed two distinct levels of intracellular HCT accumulation; in Fig. 3b, HCT-bright cells are indicated by white arrows. To determine *k*_off_, we resuspended the cells in HCT-free media and measured a decrease in the intercellular HCT intensity. HCT-bright cells maintained their HCT signals, indicating no unbinding (orange shade in Fig. 3c). These HCT-bright cells did not grow (orange shade in Fig. 3d). Longer observation revealed that these cells failed to resume growth and ultimately lysed. On the other hand, cells with weak HCT signals exhibited rapid HCT unbinding and active growth (Fig. 3c, d). While there is intrinsic heterogeneity in cell growth (in the absence of HCT), this heterogeneity is small (<30%)^54,55^. In fact, we did not observe non-growing cells in the absence of HCT.

When we analyzed these data to quantify the *k*_off_ and *λ* of individual cells, we found that the *k*_off_ and *λ* were correlated and exhibited a bimodal distribution (Fig. 3e, f). To demonstrate the effects of internal HCT accumulation on gene expression at single-cell resolution, we repeated the experiment with a strain that harbors a fluorescent *mCherry* gene. We observed that HCT-bright cells did not express the fluorescent protein whereas HCT-weak cells expressed the protein (Supplementary Video 1). This agrees with our previous data that high HCT levels inhibit gene expression (Supplementary Fig. 2), although the latter data were collected at the population level. The fact that some cells were able to keep the internal HCT level low and actively grew reveals that they were resistant to the HCT treatment. When we determined the fraction of this subpopulation, we found that the fraction was high up to 1 μM HCT but decreased at higher HCT concentrations (Fig. 3g).

Our findings collectively demonstrate the co-existence of two subpopulations with distinct unbinding kinetics and growth rates, supporting our hypothesis. We next repeated this single-cell experiment with two commercially available drugs that target DNA and exhibit anti-microbial activities, netropsin and berenil^56–58^. We again observed two distinct subpopulations (Supplementary Video 2 and 3).

## Discussion

Recent survey results show that approved drug molecules generally exhibit slow unbinding^44,45^. The importance of slow unbinding is logical, because drugs exert their effects only when bound to their targets. Additional beneficial effects of slow unbinding were proposed, e.g., target selectivity^59^ or enhanced cidality^60^. This critical effect of *k*_off_ is well supported by our study as well.

Various intracellular conditions could affect the apparent unbinding rate *k*_off_ that we reported in this study, e.g., pH, reaction volume, non-specific binding to other intracellular components. In particular, we found transcription has critical effects on *k*_off_. As a result, *k*_off_ is not a fixed value but is variable in cells, which then manifests as heterogeneous activities of the inhibitor at the single-cell level. The unbinding of inhibitors is very slow in some cells, which leads to tight inhibitor-target complex formation, and thus strong inhibition of cell growth (and vice versa in other cells). Interestingly, the heterogeneous effect of antibiotics on cells have been observed for a wide range of antibiotics^61–63^. Several studies found that this heterogeneous effect could be caused by the variation in the expression of genes that confer resistance to antibiotics^64–66^. For example, our previous study has shown that as translation inhibitors repress the action of ribosomes at sub-lethal concentrations, cells devote more resources to produce additional ribosomes, leaving little for expressing resistance genes, which then results in heterogenous expression of the resistance genes^64^. Our finding suggests that the variation in *k*_off_ could be another mechanism for the heterogenous effect of antibiotics.

Our findings have broad implications to drug discovery. Target-based in vitro assay of small-molecule inhibitors is revolutionizing the drug discovery program^3,4^. However, the discrepancy between the assay results and therapeutic efficacy has been frequently observed^5,6^. Precisely understanding what causes this discrepancy is critical to enhancing the predictive power in the drug discovery process. Our study reveals one mechanism that could contribute to this discrepancy, i.e., coupling between unbinding and cell physiology.

Given the important role of *k*_off_ in drug efficacy and based on our observation that *k*_off_ is not a fixed value but can vary, is it possible to manipulate *k*_off_ to improve drug efficacy? We tested this possibility in our proof-of-principle study (Supplementary Fig. 6). We decreased the *k*_off_ by treating cells with a low dose of a transcription inhibitor, rifampicin. With this treatment, an otherwise growth-permissive HCT concentration became growth-inhibiting, indicating enhanced inhibitory effects of HCT (Supplementary Fig. 6). Therefore, our study opens a door for a novel therapeutic strategy to improve drug efficacy.

## Methods

### Bacterial strains and culture conditions

E. coli K-12 NCM3722^67–69^ was grown in Neidhart’s MOPS minimal media^70^ with glucose and NH_4_Cl as the carbon and nitrogen sources. NCM3722 harboring *km*::T:_*ptet*_*:mCherry*, *sp*:*P*_*con*_*-TetR-LacIq*(*attB*) (NMK104) was used for measurement of mCherry expression under the microscope. NMK80, a NCM3722 harboring *P*_*tet*_*-lacZ* (TetR is constitutively expressed in this strain) was used for β-galactosidase assay^71^. To prepare experimental cultures, cells were taken from −80 °C stocks and streaked on a LB plate. Single colony was inoculated in 2 mL LB medium and grown at 37 °C with constant agitation at 250 rpm in a water bath (New Brunswick Scientific). To monitor growth, the optical density (OD_600_) of the culture was measured using a Genesys20 spectrophotometer (Thermo-Fisher) with a standard cuvette (16.100-Q-10/Z8.5, Starna Cells Inc). Before cells entered stationary phase, cells were inoculated into 5 mL MOPS minimal medium at very low densities (typically lower than the OD_600_ of ~0.0001) and cultured them overnight (pre-culture). Next morning, the pre-culture was diluted in pre-warmed, 5 mL MOPS minimal medium (experimental culture) to the OD_600_ of ~0.01 and allowed to grow exponentially to desired OD_600_.

### Hoechst 33342 unbinding rate measurement

To determine unbinding rate of Hoechst 33342 (HCT), fluorescence intensity of single cells was measured using a microscope. Experimental cultures in MOPS minimal media were prepared as described above. When a culture reached to the OD_600_ ~ 0.025, HCT was added and incubated for 45 min. For HCT concentrations >5 μM, HCT was added at the OD_600_ ~0.04. To measure the unbinding rate of HCT under the influence of antibiotics (rifampicin 10 μg/mL, thiolutin 5 μg/mL), drugs were added at same time as HCT was added to the cultures.

After incubation with HCT, 5 μL aliquot of the culture was placed between a no. 1.5 cover glass and 1mm-thick 1.5% agarose pad^72^ in MOPS minimal media with HCT (with appropriate drugs) and imaged under the microscope. See below for details of microscopy procedures. Fluorescence intensity of these cells was recorded (time 0 min). Then, the culture was centrifuged at 3500 rpm at 37 °C for 5 min and resuspended in fresh media without HCT (with appropriate drugs). Resuspended cells were incubated at 37 °C with agitation in water bath. 5 μL aliquot of the culture was placed between a no. 1.5 cover glass and 1mm-thick 1.5% agarose pad in media without HCT and imaged under the microscope.

HCT unbinding rate was also measured by following individual cells over time (Fig. 3). Cells were prepared as described above with HCT 5 μM. After incubation, cells were placed between agarose pad in media without HCT and a coverslip of 35 mm glass bottom dish (Cellvis) and imaged under the microscope over time. See the Supplementary Table 1 for all the chemicals and concentrations used.

### HCT unbinding rate measurement with chloramphenicol

Cells were prepared as described above. At the OD_600_ ~0.025, 1 μM of HCT and 20 μg/mL of chloramphenicol was added. After 45 min of incubation, 5 μL aliquot of culture was transferred onto a no. 1.5 cover glass, sandwiched by 1mm-thick 1.5% agarose pad^72^, and imaged with a microscope. For time-lapse microscopy to measure the unbinding rate of HCT in individual cells, 1.5% agarose pad in 35 mm glass bottom dish (Cellvis) was used.

### HCT binding rate measurement

Cells were prepared as described above. HCT was added to a culture when OD_600_ reached to ~0.1. Fluorescence images were acquired periodically by transferring 5 μL aliquot of culture between a no. 1.5 cover glass and 1mm-thick 1.5% agarose pad in media with HCT and using a microscope.

### Time-lapse imaging of mCherry expression

NCM3722 harboring *km*::T:_*ptet*_*:mCherry*, *sp*:*P*_*con*_*-TetR-LacIq*(*attB*) (NMK104) was used to visualize the influence of DNA binding drugs on gene expression. Cells were grown as described above. When OD_600_ reached to ~0.05, 10 μM HCT, 15 μg/mL netropsin, or 30 μg/mL berenil was added to the culture. After incubation with drugs (1 h, 4.5 h, and 4 h for HCT, netropsin, and berenil, respectively), cells were plated between a cover glass and 1.5% agarose in MOPS minimal media with 100 ng/mL aTc on 35 mm glass bottom dish (Cellvis). Time-lapse images were captured using a microscope. mCherry fluorescence was detected using a TRITC filter cube.

### β-galactosidase assay

NCM3722 harboring *P*_*tet*_*-lacZ* (NMK80, TetR is constitutively expressed in this strain) was used^71^. For a control (HCT-free), anhydrotetracycline (aTc) was added to a culture at OD_600_ of ~0.1 to the final concentration of 100 ng/mL. For HCT treatment, HCT was added at OD_600_ of ~0.05 and then after 3 hr of incubation, aTc was added to the final concentration of 100 ng/mL. Samples were rapidly frozen in ethanol dry ice bath and stored at −80 °C. The β-galactosidase assay was carried out as described^73^, with some modification. Samples were thawed and kept on ice. Cells were lysed in a mixture of Z-buffer, SDS, and β-mercaptoethanol (final volume of 1 mL). 50 μL of chloroform was added, and solutions were vortexed for 15 sec and incubated at 37 °C in a water bath for 5 min. 200 μL of pre-warmed ONPG (4 mg/mL) was added to the solution. The reaction was stopped by adding 500 μL of 1 M NaCO_3_ when the color of solution turned to light yellow. 800 μL of reaction was spun down at 10k rpm for 3 min and optical density at 420 nm and 550 nm were measured using a Genesys20 spectrophotometer (Thermo-Fisher) with a standard cuvette (16.100-Q-10/Z8.5, Starna Cells Inc).

### Fluorescence microscopy and image analysis

All image acquisition was done using an inverted fluorescence microscope (Olympus IX83) with an oil immersion phase-contrast 60× objective seated inside an incubator chamber (InVivo Scientific) pre-warmed to 37 °C and MetaMorph software (Molecular Devices). HCT fluorescence was measured using a DAPI filter cube. Images were captured using a Neo 5.5 sCMOS camera (Andor).

Fluorescence intensity was obtained with MicrobeJ (5.13 l (4))^74^, a plug-in for the ImageJ software. MicrobeJ can automatically segment cell boundaries from phase-contrast microscope images and apply the binary masks from the segmentation to measure fluorescence intensities (in mean gray value) inside and outside the cells. The latter (background) is subtracted from the former to determine intracellular fluorescence signals. Autofluorescence of cells was determined by measuring fluorescence intensity in the absence of HCT. Reported intensities are the results of subtracting autofluorescence from intracellular fluorescence signals.

### Statistics and reproducibility

At least two biological replicates were conducted for all experiments, and all data were reproducible. For single cell analysis, data points were determined from averaging 100~200 cells. For Figs. 1b, d, 2a, b, and 3g, means and raw data points were plotted. For Fig. 1a, c and 3b–f, data points from a single experiment were plotted to show a typical pattern, and the observed pattern was confirmed in the other biological replicate. For Fig. 1a, one-tailed *t*-test was conducted to show a statistically significant difference of the growth rates between HCT 1 and 3 μM (*P*-value = 0.0325).

### Reporting summary

Further information on research design is available in the Nature Portfolio Reporting Summary linked to this article.

## Supplementary information

Supplementary Information

Description of Additional Supplementary Files

Sup Mov 1

Sup Mov 2

Sup Mov 3

Sup Mov 4

Sup Mov 5

Supplementary Data

Reporting Summary

## Data Availability

The datasets are provided as Supplementary Data.
