# Supplementary material for: Dissociation kinetics of small-molecule inhibitors in Escherichia coli is coupled to physiological state of cells
Source: Commun Biol. 2023 Feb 25;6:223. doi: 10.1038/s42003-023-04604-9 (PMC9968327; doi:10.1038/s42003-023-04604-9)
Supplement: Supplementary file 1 — Supplementary Information [file 42003_2023_4604_MOESM1_ESM.pdf]

SUPPLEMENTARY MATERIALS

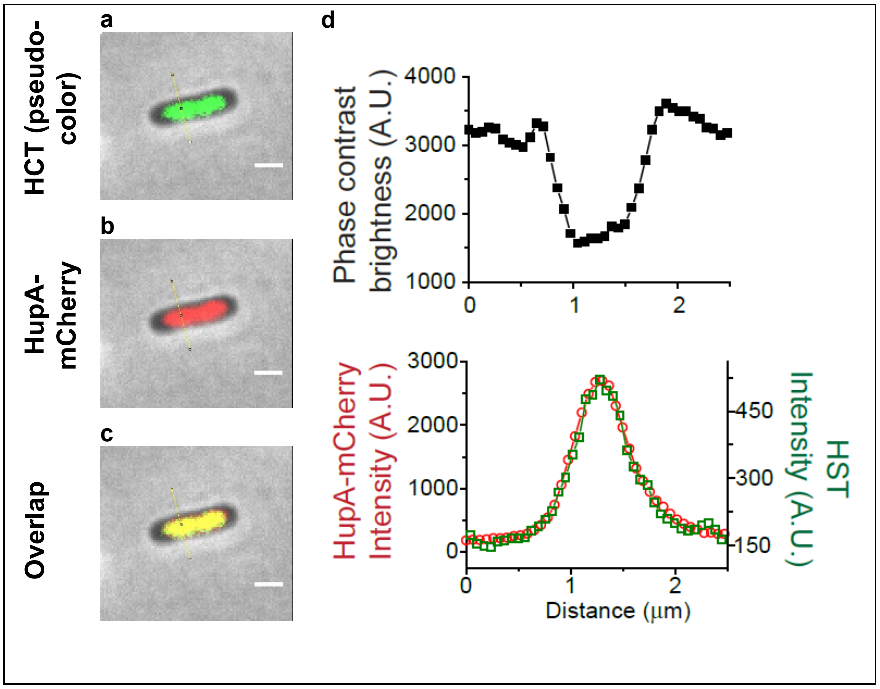

Supplementary Fig. 1. HCT fluorescence signal represents DNA binding

To test that HCT signal is predominantly from its binding to DNA, we performed a co-localization experiment. **a).** HCT fluorescence (green) in *E. coli*. Although HCT emits blue fluorescence, it is displayed in green here because green is more visible. Scale bar represents 1μm. **b).** We fluorescently labeled the nucleoid (the region containing DNA) with mCherry-tagged nucleoid-associated protein HupA (HupA-mCherry) [1]. **c).** HCT signal spatially overlapped with, and was confined to, the nucleoid (green + red = yellow). **d).** Quantitative analysis of the images. Phase contrast brightness shows the cell area. HCT and mCherry signals exhibit identical spatial profiles. This result demonstrates that intracellular HCT fluorescence comes predominantly from its binding to DNA. The plot shows representative data from a single experiment. A biological replicate was conducted to confirm that the pattern was reproducible.

a) **b)**

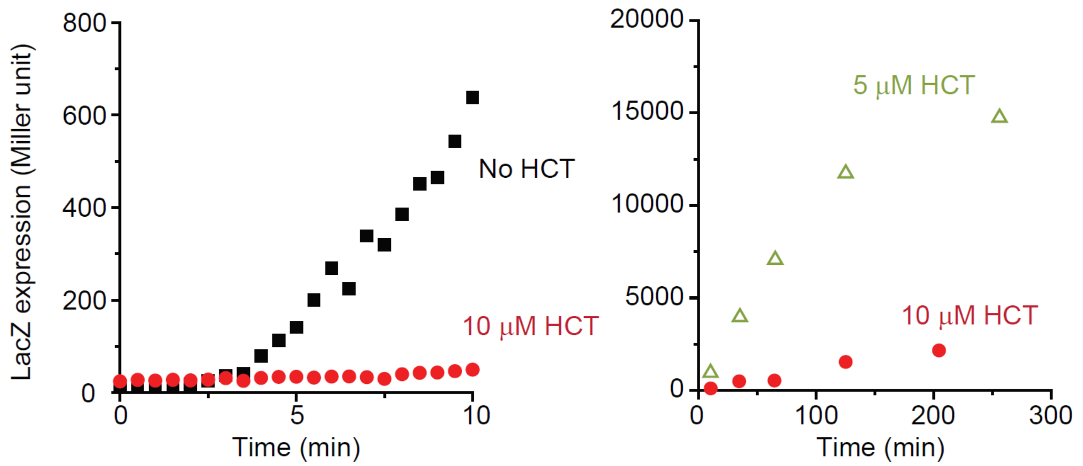

b)

Supplementary Fig. 2. HCT inhibits gene expression.

NMK80 harbors P_{tet}-*lacZ* (TetR is constitutively expressed in this strain) [2]. In this construct, LacZ expression can be activated by an inducer, anhydrous tetracycline (aTc).

a). We cultured cells with and without 10 μM HCT, added 100 ng/ml aTc and measured the LacZ expression using a β-galactosidase assay. 10 μM HCT repressed the LacZ expression (red circles). **b).** Long-term measurement of LacZ expression. LacZ expression remains low at 10 μM HCT. The green symbols show LacZ expression with 5 μM HCT. The plot shows representative data from a single experiment. A biological replicate was conducted to confirm that the pattern was reproducible.

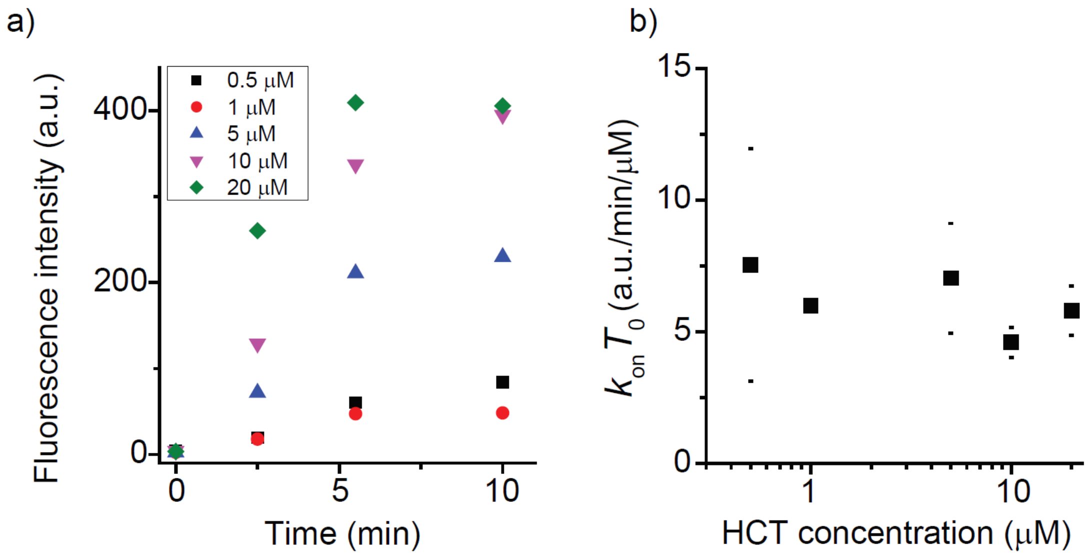

Supplementary Fig. 3. k_{on} is relatively constant over various HCT concentrations.

a). Accumulation of HCT during growth in bulk culture. Intracellular HCT intensities of ~150 cells were measured and averaged for each data point. The plot shows representative data from a single experiment. A biological replicate was conducted to confirm that the pattern was reproducible.

b). We estimated the binding kinetic rate k_{on} in the following way. The binding and unbinding between HCT and DNA can be described as,

$$\frac{d[H{\cdot}T]}{dt} = k_{on}([T_0] - [H \cdot T]) \cdot [H] - k_{off}[H \cdot T] \qquad \text{Eq. S1}$$

where $[H]$ is the HCT concentration, $[T_0]$ is the total concentration of the HCT target binding site, and $[H{\cdot}T]$ is the concentration of HCT-target complex. Immediately after HCT was introduced, $[H{\cdot}T]$ is low and we approximate Eq. S1 as

$$\frac{d[H{\cdot}T]}{dt} = k_{on} \cdot [T_0] \cdot [H], \qquad \text{Eq. S2}$$

and thus

$$[H{\cdot}T] = k_{on} \cdot [T_0] \cdot [H] \cdot t. \qquad \text{Eq. S3}$$

Therefore, $[H{\cdot}T]$ should initially increase linearly immediately after HCT addition, which agrees with the observed linear increase in the HCT fluorescence intensity up to ~5 min (left panel). We then calculated the slope of this linear increase and divided it by the HCT concentration, which yields $k_{on} \cdot [T_0]$; see Eq. S3. This quantity is plotted in the right panel, which shows that its value is unchanged over various HCT concentrations. Here, $[T_0]$ is the total concentration of the HCT binding site (DNA minor groove), which also should be unchanged. Therefore, k_{on} is relatively constant over the range of HCT concentration characterized. Small lines indicate the data from two biological repeats, and the solid squares indicate their mean.

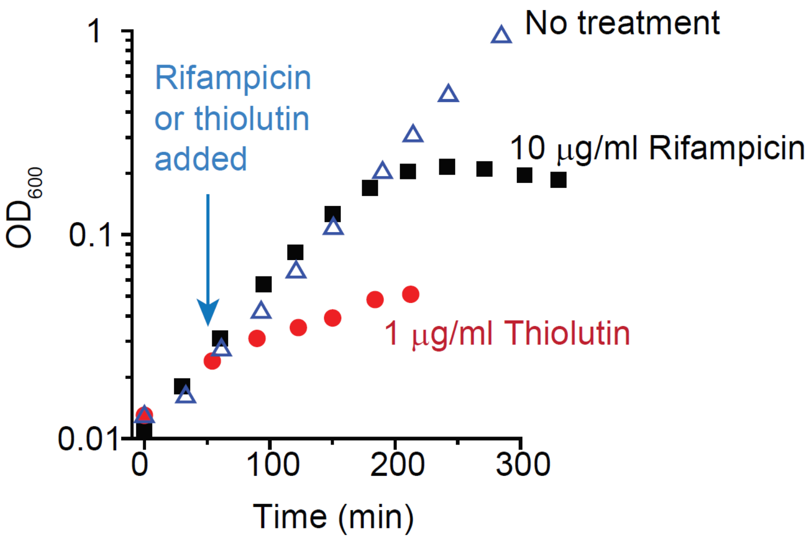

Supplementary Fig. 4. Effects of rifampicin and thiolutin on cell growth.

Cells were treated with 10 μg/mL of rifampicin and 1 μg/mL thiolutin in bulk culture. The plot shows representative data from a single experiment. A biological replicate was conducted to confirm that the pattern was reproducible.

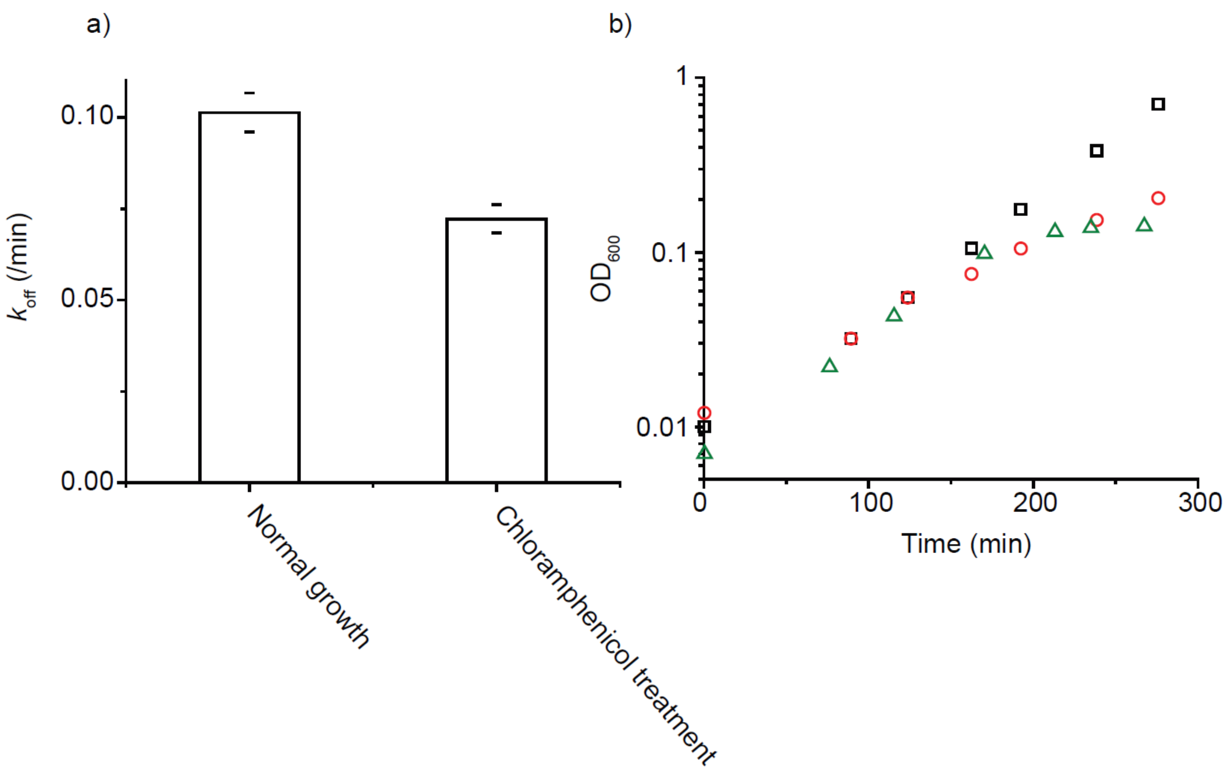

Supplementary Fig. 5. Effects of chloramphenicol on HCT unbinding

a). Comparing k_{off} for 0 and 20 µg/ml of chloramphenicol. Small lines indicate the data from two biological repeats, and the columns indicate their mean.

b). Growth curve. Black square, control with HCT 0 µM; Red circles, HCT 5 µM was added at 130 min; Green triangles, 20 µg/ml of chloramphenicol was added at 170 min, which led to complete growth inhibition. The plot shows representative data from a single experiment. A biological replicate was conducted to confirm that the pattern was reproducible.

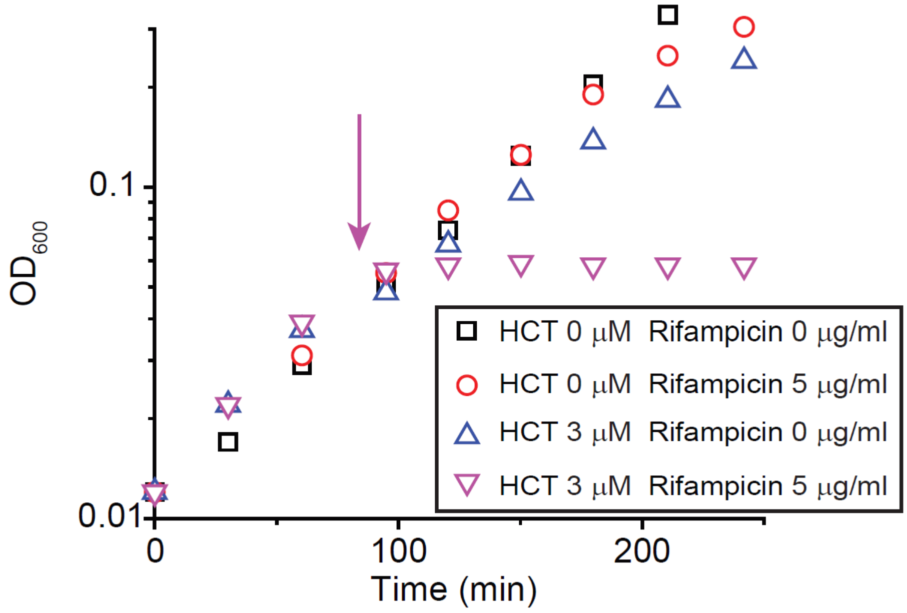

Supplementary Fig. 6 Synergistic effects of HCT and rifampicin

3 µM HCT or 5 µg/ml rifampicin alone had only marginal effects on cell growth. When administered together, however, they completely inhibited cell growth. The plot shows representative data from a single experiment. A biological replicate was conducted to confirm that the pattern was reproducible.

Supplementary Table 1A

Ingredients used in the MOPS minimal media are provided below.

Ingredient	Concentration	Manufacturer	Catalog
MOPS	80 mM	Sigma Aldrich	1132-61-2
Tricine	4 mM	Sigma Aldrich	5704-04-1
$FeSO_4$	0.01 mM	Acros Organics	7782-63-0
K_2SO_4	0.276 mM	Sigma Aldrich	7778-80-5
$CaCl_2$	0.05 mM	Sigma Aldrich	10043-52-4
$MgCl_2.6H_2O$	0.523 mM	Sigma Aldrich	7791-18-6
K_2HPO_4	0.132 mM	Sigma Aldrich	7758-11-4
NaCl	50 mM	Sigma Aldrich	7647-14-5
NH_4Cl	10 mM	Sigma Aldrich	12125-02-9
Glucose	10 mM	Sigma Aldrich	50-99-7
Micronutrients			
$(NH_4)_6Mo_7O_{24}*4H_2O$	3 nM	Acros Organics	12054-85-2
H_3BO_3	400 nM	Milipore	10043-35-3
$CoCl_2.6H_2O$	30 nM	Acros Organics	7791-13-1
$CuSO_4$	10 nM	Acros Organics	7758-99-8
$MnCl_2$	80 nM	Acros Organics	13446-34-9

$ZnSO_4.7H_2O$	10 nM	Acros Organics	7446-20-0

Supplementary Table 1B

Other chemicals used in the experiments

Chemical	Manufacturer	Catalog
bisBenzimide Hoechst 33342 trihydrochloride	Sigma Aldrich	875756-97-1
Diminazene aceturate	Sigma Aldrich	908-54-3
Rifampicin	Bio Basic	13292-46-1
Netropsin dihydrochloride	Enzo Life Sciences	89149-986
Thiolutin	Sigma Aldrich	87-11-6
Chloramphenicol	Sigma Aldrich	56-75-7

Supplementary Movie

Supplementary Movie 1. Cells harboring P_{tet}:*mCherry* (TetR was constitutively expressed) was pre-incubated with 1 µM HCT for 1 hour and then transferred to fresh MOPS 1.5% agarose pad with 10 mM glucose, 10 mM NH_4Cl, and 100 ng/mL aTc. Red fluorescence indicates mCherry expression. Green fluorescence indicates HCT accumulation.

Supplementary Movie 2. Cells harboring P_{tet}:*mCherry* (TetR was constitutively expressed) was pre-incubated with 15 µg/mL netropsin for 4.5 hours and then transferred to fresh MOPS 1.5% agarose pad

with 10 mM glucose, 10 mM NH_4Cl, and 100 ng/mL aTc. Red fluorescence indicates mCherry expression.

Supplementary Movie 3. Cells harboring P_{tet}*:mCherry* (TetR was constitutively expressed) was pre-incubated with 30 μg/mL berenil for 4 hours and then transferred to fresh MOPS 1.5% agarose pad with 10 mM glucose, 10 mM NH_4Cl, and 100 ng/mL aTc. Red fluorescence indicates mCherry expression.

Supplementary Movie 4. Cells were pre-incubated with 1 μM HCT for 1 hour and then with 10 μg/ml rifampicin for 3 hours. Cells were transferred to fresh MOPS 1.5% agarose pad with 10 mM glucose, 10 mM NH_4Cl, and 10 μg/ml rifampicin, but no HCT. Green fluorescence indicates HCT intensity. Panel a shows a typical time-lapse sequence. There were occasionally bright cells, e.g., a cell in the lower right-hand corner, which was zoomed in in panel c with the brightness of images adjusted. Due to their high fluorescence intensity, the fluorescence intensity of other cells was not visible in panel a. In panel b, the brightness of images was adjusted to visualize the HCT fluorescence of cells with low initial intensity.

Supplementary Movie 5. Cells were pre-incubated with 1 μM HCT for 1 hour and then with 20 μg/ml chloramphenicol for 1 hour. Cells were transferred to fresh MOPS 1.5% agarose pad with 10 mM glucose, 10 mM NH_4Cl, and 20 μg/ml chloramphenicol, but no HCT. Green fluorescence indicates HCT intensity.

Supplementary Data. The file includes numerical data for main figures.

References

1 Marceau, A. H., Bahng, S., Massoni, S. C., George, N. P., Sandler, S. J., Marians, K. J. & Keck, J. L. Structure of the SSB–DNA polymerase III interface and its role in DNA replication. *The EMBO journal* **30**, 4236-4247 (2011).

2 Iyer, S., Le, D., Park, B. R. & Kim, M. Distinct mechanisms coordinate transcription and translation under carbon and nitrogen starvation in Escherichia coli. *Nature Microbiology* **3**, 741–748 (2018).
