# Supplementary material for: Dissociation kinetics of small-molecule inhibitors in Escherichia coli is coupled to physiological state of cells
Source: Commun Biol. 2023 Feb 25;6:223. doi: 10.1038/s42003-023-04604-9 (PMC9968327; doi:10.1038/s42003-023-04604-9)
Supplement: Supplementary file 2 — Description of Additional Supplementary Files [file 42003_2023_4604_MOESM2_ESM.pdf]

Description of Additional Supplementary Files

File name: Supplementary Data 1

Description: Numerical data used to plot figures.

File name: Supplementary Movie 1

Description: Time lapse movie of cells harboring Ptet:mCherry pre-incubated with 10 uM HCT on MOPS agarose media with 100 ng/mL aTc

File name: Supplementary Movie 2

Description: Time lapse movie of cells harboring Ptet:mCherry pre-incubated with 15 ug/mL netropsin on MOPS agarose media with 100 ng/mL aTc

File name: Supplementary Movie 3

Description: Time lapse movie of cells harboring Ptet:mCherry pre-incubated with 30 ug/mL berenil on MOPS agarose media with 100 ng/mL aTc

File name: Supplementary Movie 4

Description: Time lapse movie of wild type cells pre-incubated with 1 uM HCT and 10 ug/mL rifampicin on MOPS agarose media with 10 ug/mL rifampicin

File name: Supplementary Movie 5

Description: Time lapse movie of wild type cells pre-incubated with 1 uM HCT and 20 ug/mL chloramphenicol on MOPS agarose media with 20 ug/mL chloramphenicol
